# Artificial intelligence for skin cancer detection and classification for clinical environment: a systematic review

**DOI:** 10.3389/fmed.2023.1305954

**Published:** 2024-01-08

**Authors:** Brunna C. R. S. Furriel, Bruno D. Oliveira, Renata Prôa, Joselisa Q. Paiva, Rafael M. Loureiro, Wesley P. Calixto, Márcio R. C. Reis, Mara Giavina-Bianchi

**Affiliations:** ^1^Imaging Research Center, Hospital Israelita Albert Einstein, São Paulo, Brazil; ^2^Electrical, Mechanical and Computer Engineering School, Federal University of Goiás, Goiânia, Brazil; ^3^Studies and Researches in Science and Technology Group (GCITE), Federal Institute of Goiás, Goiânia, Brazil

**Keywords:** Skin cancer, artificial intelligence, melanoma, detection, classification, feature extraction

## Abstract

**Background:**

Skin cancer is one of the most common forms worldwide, with a significant increase in incidence over the last few decades. Early and accurate detection of this type of cancer can result in better prognoses and less invasive treatments for patients. With advances in Artificial Intelligence (AI), tools have emerged that can facilitate diagnosis and classify dermatological images, complementing traditional clinical assessments and being applicable where there is a shortage of specialists. Its adoption requires analysis of efficacy, safety, and ethical considerations, as well as considering the genetic and ethnic diversity of patients.

**Objective:**

The systematic review aims to examine research on the detection, classification, and assessment of skin cancer images in clinical settings.

**Methods:**

We conducted a systematic literature search on PubMed, Scopus, Embase, and Web of Science, encompassing studies published until April 4th, 2023. Study selection, data extraction, and critical appraisal were carried out by two independent reviewers. Results were subsequently presented through a narrative synthesis.

**Results:**

Through the search, 760 studies were identified in four databases, from which only 18 studies were selected, focusing on developing, implementing, and validating systems to detect, diagnose, and classify skin cancer in clinical settings. This review covers descriptive analysis, data scenarios, data processing and techniques, study results and perspectives, and physician diversity, accessibility, and participation.

**Conclusion:**

The application of artificial intelligence in dermatology has the potential to revolutionize early detection of skin cancer. However, it is imperative to validate and collaborate with healthcare professionals to ensure its clinical effectiveness and safety.

## 1 Introduction

The role of technology and artificial intelligence has gained increasing prominence in the field of dermatology. Techniques such as convolutional neural networks and image processing have been extensively examined for their capacity to identify specific features in skin lesion images, with the potential to aid in the recognition of suspicious lesions and the diagnosis of conditions like melanoma.

Skin cancer is the most common form of cancer worldwide (1). Over the past decade, there has been a concerning 27% increase in the annual diagnosis of invasive melanoma cases (2). Alarmingly, more than 5,400 people die from non-melanoma skin cancer every month (3). In the United States alone, the annual financial burden of treating skin cancer is estimated at a staggering *US$*8.1 billion, with approximately *US$*4.8 billion allocated to non-melanoma skin cancer and *US$*3.3 billion to melanoma (4). Among skin cancer types, basal cell carcinoma ranks as the most common, followed by squamous cell carcinoma and melanoma, which stands out as the most aggressive and lethal type of skin cancer (5, 6). Merkel cell carcinoma also stands out among aggressive tumors (7). These tumors can arise anywhere on the body but are frequently observed in regions more exposed to the sun, including the face, neck, arms, and hands. Thus, there is an imperative need for sustained efforts to promote awareness and prevention of skin cancer (8–10).

Conventional techniques for detecting these diseases include patient data analysis, as well as visual and histopathological analysis of the lesions (11). Visual assessment relies on the clinical inspection of the lesion, taking into consideration factors such as its appearance, size, shape, location, and evolution. On the other hand, histopathological analysis entails the collection of a sample of the lesion for laboratory examination, typically through techniques such as biopsy. Additionally, devices like the dermatoscope are used to facilitate the examination of the lesion and the identification of features such as pigmentation, vascularity, and regression (12). Another example is the use of confocal microscopy, a technique that allows the analysis of skin layers without the need for sample collection (13, 14).

These techniques have proven effective in the detection and diagnosis of skin diseases. However, they may present limitations, including subjectivity in visual analysis and the need for invasive sample collection procedures. Confocal microscopy incurs high financial costs and is relatively inaccessible to medical professionals, even among specialists.

It is also important to highlight that diagnosing these diseases poses a significant challenge to the healthcare system, especially in regions lacking specialized professionals or adequate equipment for skin lesion identification (15, 16). An alternative approach involves initial screening by general practitioners, who may not always possess the necessary training for early skin cancer detection (17).

The implementation of Computer-Aided Diagnosis (CAD) solutions powered by Artificial Intelligence (AI) holds the potential to address some of these limitations and offer a promising alternative for accurate and non-invasive skin disease diagnosis. Existing literature suggests that AI systems can classify skin cancers competently on par with dermatologists. Notably, the diagnostic capabilities of the dermatologist vary based on experience, i.e., it is not a uniform basis of reference. Moreover, studies highlight the feasibility of leveraging mobile devices equipped with neural networks to broaden the access of dermatological expertise, offering low-cost access to vital diagnostic care (18, 19).

While numerous solutions are being developed for skin cancer detection and classification, those are usually not evaluated and validated in real clinical settings, which limits their practical applicability. The review study conducted by Goyal et al. (20) provides an updated assessment of the performance of artificial intelligence algorithms in skin cancer classification and diagnosis. It also delves into the challenges faced by these systems and future opportunities to enhance of dermatologists' diagnostic abilities through AI support.

However, for these technologies to become effective and applicable in clinical settings, several challenges must be addressed. These challenges include the need for standardization in image acquisition and processing techniques, the requirement for extensive training datasets, and the creation of robust and representative databases (20–24). Prior studies in skin cancer classification have have demonstrated restricted generalizability due to insufficient data and an emphasis on standardized tasks (19). Furthermore, it is essential to evaluate the effectiveness and safety of these tools in diverse contexts, taking into account variables such as the ethnic and genetic diversity of the population and the specific type of skin cancer under consideration, among other factors. In this regard, it is imperative for research in this field to adhere rigorously to scientific and ethical standards. Finally, it is crucial to emphasize that automated skin disease detection should not replace clinical evaluation by medical professionals but rather complement it.

The aim of this systematic review is to investigate studies focused on the detection, classification, and evaluation of skin cancer images in a clinical setting. The main approaches and challenges encountered while implementing these techniques must be identified to do this. The importance of this systematic review lies in its ability to aggregate and thoroughly examine all pertinent research in this field, thus offering a comprehensive view of the subject. In turn, researchers can assess the quality and credibility of existing studies, identify knowledge gaps, and propose innovative research directions. Furthermore, this systematic review can provide valuable information for doctors and healthcare professionals looking to harness the potential of AI in aiding the diagnosis and treatment of skin diseases.

## 2 Methods

This section outlines the methodology employed for the systematic literature review, encompassing the following stages: (i) research identification, (ii) selection, (iii) eligibility, (iv) data extraction, and (v) synthesis.

### 2.1 Step 1: study identification

First, we established the objectives and questions that frame this literature review. The primary goal of this systematic review is to highlight research involving the implementation of AI in clinical settings. Our aim is to gain insights into the methodologies employed in previous research and the outcomes achieved when using AI in this context.

For this review, we registered a protocol with the International Prospective Register of Systematic Reviews (PROSPERO) under ID CRD42023411211 on April 4, 2023, and PRISMA guidelines were followed. PROSPERO is a global registry for systematic review protocols, where researchers publish their research methods in advance. This process promotes transparency, prevents publication bias, and improves the reproducibility of studies.

The search databases used for the literature review include PubMed, Scopus, Embase, and Web of Science, and topics are analyzed using the following search terms: (“skin cancer” OR “skin lesion” OR “dermatology” OR “dermatoscopy” OR “melanoma”) AND (“artificial intelligence” OR “neural network*” OR “deep learning” OR “convolutional neural network*” OR “transfer learning” OR “machine learning” OR “Computer aided diagnostic*” OR “CAD” OR “image classification” OR “image processing” OR “Internet of things” OR “Data mining” OR “Iot”) AND (“real-time” or “real time” OR “real-world” OR “real world” OR “smartphone”) AND NOT (“Meta-Analysis” OR “Meta Analysis” OR “Systematic Review”).

### 2.2 Step 2: study selection

Secondly, we defined the search terms and established inclusion/exclusion criteria. In this literature review, we used the terms highlighted in the previous section, with the sole restriction being the inclusion of journal articles and conference proceedings only.

Our initial search yielded 760 results, of which 457 were identified as duplicates and therefore removed. This resulted in a pool of 303 distinct studies, which were subsequently evaluated for eligibility.

### 2.3 Step 3: study relevance and quality assessment

In the third step, we assessed the relevance and quality of the selected studies. Two authors (BCRSF and MRCR) were responsible for reading each title and abstract in order to assess the relevance and quality of each previously selected study. The criteria used to determine eligibility is as follows:

- The document's abstract presents clear objectives, methodology, and results.
- The study addresses computer-aided diagnostic solutions for skin cancer with a focus on real clinical applications.
- The study reports the accuracy, sensitivity, specificity, and/or overall accuracy of artificial intelligence systems for skin cancer.
- The study describes the development and/or validation process of the systems.
- The study provides a critical analysis of the results obtained by artificial intelligence systems and discusses their limitations and potential biases.

**Figure 1:**
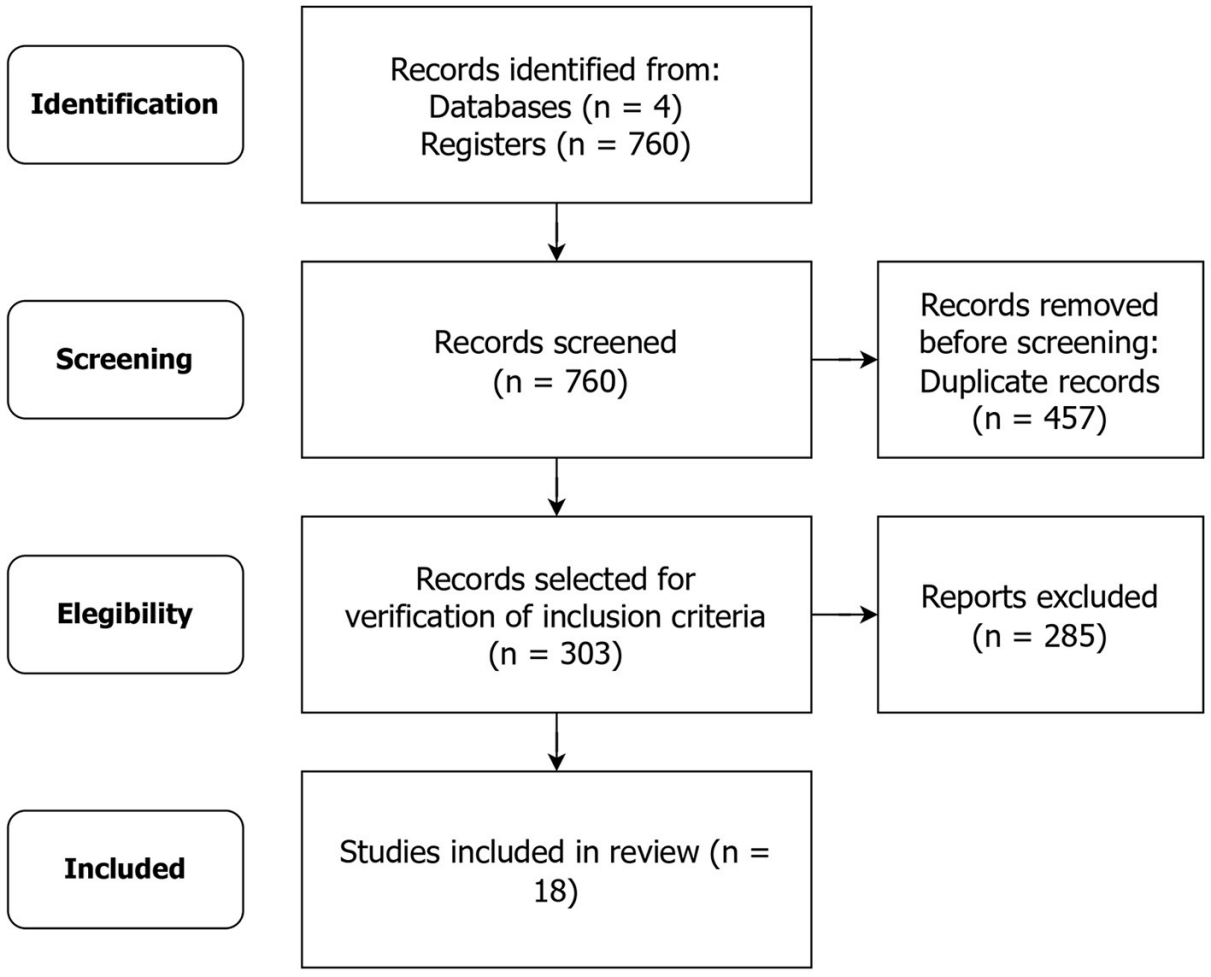
Study selection, evaluation, and inclusion (presented using the PRISMA flow diagram).

Based on the inclusion criteria stated above, a total of 282 studies were eliminated from consideration. Following a comprehensive review of the entire texts, three more studies were removed from consideration due to their limited content, which included only abstracts or incomplete texts. Ultimately, 18 studies have been retained. Figure 1 presents the study identification flowchart.

For the study, Mendeley and Rayyan tools were used.

### 2.4 Step 4: data extraction

To facilitate data extraction in our literature review, we utilized a spreadsheet to document the metadata of each selected study. The following metadata was analyzed:

1. Publication year and study objective.
2. Regarding the data used: Types of data, source, and quantity.
3. Resources used to assist in the detection and/or classification of skin lesions.
4. Technique for the detection and/or classification of skin lesions.
5. Study function.
6. Key findings and study perspectives.
7. Information regarding ethnic and genetic diversity of the population.
8. Information regarding system accessibility and availability.
9. Relationship and/or involvement of dermatologists and other medical professionals.

### 2.5 Step 5: data synthesis

The concluding phase of our study encompasses data synthesis, which was subdivided into two key steps. Initially, we conducted a systematic analysis of the raw data obtained through the literature review process. Subsequently, we compiled metadata pertaining to the articles chosen in our literature review.

## 3 Results and discussion

This section outlines the results obtained through the search strategies describes in the methodology.

### 3.1 Descriptive analysis

The first section of our analysis pertains to descriptive information. As part of this analysis, we examined the objectives of the selected studies.

The primary objective of all the mentioned studies is to develop, implement, and/or validate systems for the detection, diagnosis, and classification of skin cancers, particularly melanoma, using mobile devices or computers. These systems aim to improve the early detection of skin lesions and enhance diagnostic accuracy, assisting healthcare professionals and providing more accessible and efficient screening for patients. Furthermore, they explore the use of advanced techniques such as image processing, pattern recognition, and deep learning to automate the analysis process and deliver real-time results.

### 3.2 Data scenario

For our study, it is of utmost importance to analyze the quality and representativeness of the data, given that these factors play a critical role in developing reliable algorithms and models for skin lesion diagnosis. Diversity in data sources is key to ensure broader model generalization since different sources can provide specific and varied information about the lesions. Furthermore, the availability of large datasets containing hundreds of thousands of images can be extremely advantageous in creating more robust and accurate machine learning models. Table 1 presents important information about the data from each article. The study information is listed in chronological order based on the publication date.

**Table 1:** Overview of studies on skin cancer image analysis: data type, origin, and quantity by year and author.

**References**	**Data type**	**Data origin**	**Total amount of initial data**
Ramlakhan and Shang (25)	Clinical (macroscopic) images acquired by mobile device.	Randomly collected images from the internet.	Dataset of 37 images of benign skin lesions and 46 images of malignant lesions.
Afifi et al. (26)	Clinical (macroscopic) images.	Not specified how they are acquired.	Dataset of 356 images, including 168 melanoma images.
Thissen et al. (27)	Clinical (macroscopic) images acquired by mobile device.	SkinVision application.	Dataset of 341 images of melanocytic and non-melanocytic lesions, with 239 undergoing histopathological examination, while the other 102 lesions were clinically diagnosed as benign and not removed.
Roy et al. (28)	Dermoscopic images.	PH2 database.	Dataset of 200 dermoscopic images from the PH2 database, including 80 common nevi, 80 atypical nevi, and 40 melanomas.
Alizadeh and Mahloojifar (29)	Dermoscopic images.	ISIC Database.	Dataset of 150 dermatoscopy images from the ISIC website, consisting of 75 images for non-melanoma lesions and 75 images for melanoma lesions.
Fujisawa et al. (30)	Clinical (macroscopic) images from digital cameras.	Patient data from the University of Tsukuba Hospital from 2003 to 2016.	Dataset of 6,009 images from 2,296 patients, including 14 diagnoses, both malignant and benign conditions.
Udrea et al. (31)	Clinical (macroscopic) images acquired by mobile device.	Data obtained from the University Hospital of Munich and a hospital in Eindhoven, funded by SkinVision BV.	Dataset of 131,873 images acquired from 31,449 users of the app. It included 285 histopathologically validated skin cancer cases, including 138 malignant melanomas.
Bakheet and Al-Hamadi (32)	Dermoscopic images.	PH2 public dataset.	Dataset of 200 images, including 40 malignant and 160 benign lesions.
Abbas (33)	Dermoscopic images.	Various public and private sources, including EDRA-CDROM, ISIC, DermNet, and PH2.	Total of 2,200 dermatoscopy images, including 1,100 malignant melanomas (MM) and 1,100 benign tumors.
Bakheet and El-Nagar (34)	Dermoscopic images.	PH2 public dataset.	Dataset of 200 images, including 80 common nevi, 80 atypical nevi, and 40 melanomas.
Dulmage et al. (35)	Clinical (macroscopic) images.	Images collected by primary care professionals.	Dataset of 76,926 images annotated by dermatologists from the VisualDx privately curated image database, focusing on lesion morphology analysis.
Pangti et al. (36)	Clinical (macroscopic) images.	Raw images from public databases (http://www.hellenicdermatlas.com/en and http://www.danderm.dk/atlas), as well as images from dermatologists in India.	Initial total dataset of 17,718 images. Of these, 310 images were discarded during preprocessing due to poor resolution or multiple lesions. Of the remaining 17,408 images, 1,990 images belonged to the non-specific category, and 15,418 images fell within the 40 selected disease categories.
Giavina-Bianchi et al. (17)	Clinical (macroscopic) and dermoscopic images.	Clinical Model: Teledermatology Project. Dermatoscopic Model: ISIC2019 and PH2 datasets.	Clinical Model: Dataset of 14,000 images belonging to seven classes. Dermatoscopic Model: Dataset of 26,342 images.
Francese et al. (37)	Clinical (macroscopic) images acquired by mobile device.	Despite lack of specification, there is an assumption that the images originate from the authors.	Dataset of 8,000 melanoma or non-melanoma images.
Felmingham et al. (24)	Dermoscopic images.	Two Australian tertiary centers: Skin Health Institute and Alfred Hospital in Melbourne, Australia.	The study aims to recruit 220 participants and provide a minimum of three lesions per participant for final analysis.
Sangers et al. (38)	Clinical (macroscopic) images acquired by mobile device.	University hospital in the Netherlands.	Dataset of 785 skin lesion images, including 418 suspected lesions and 367 benign lesions used as controls.
Jahn et al. (39)	Clinical (macroscopic) images acquired by mobile device.	Dermatology Department at University Hospital Basel, Switzerland.	Dataset of 1,204 pigmented skin lesions.
Kränke et al. (40)	Clinical (macroscopic) images acquired by mobile device.	Tertiary reference center in Graz, Austria.	Dataset of 1,171 images.

The analysis of the studies reveals a remarkable diversity of approaches in the diagnosis of skin tumors, with a significant emphasis on the detection of melanoma and other dermatological conditions. Among these research studies, there is a notable convergence in the preference for the use of clinical (macroscopic) images and/or dermoscopic images for analysis. The choice of these images demonstrates a consensus in the scientific community regarding the importance of this data in developing more effective and accessible diagnostic methods.

In the context of the types of data employed, Roy et al. (28) and Alizadeh and Mahloojifar (29) used dermoscopic images from established databases like PH2 and ISIC, adding to the reliability of the results. Meanwhile, Dulmage et al. (35) relies on clinical images collected by healthcare professionals, reflecting real-world conditions.

The discrepancy in the size of datasets is evident, with some studies using relatively small datasets, such as Ramlakhan and Shang (25) and Afifi et al. (26), which have 83 and 356 images, respectively. This limitation in size may restrict the models' capacity for generalization and accuracy. On the other hand, Udrea et al. (31) and Pangti et al. (36) present massive datasets containing 131,873 and 17,408 images, respectively. This provides a more solid foundation for model generalization and learning. Furthermore, Thissen et al. (27) works with a dataset of 341 images, which is still considerably limited compared to the larger datasets. This difference in dataset size directly impacts the models' ability to generalize, emphasizing the importance of carefully assessing effectiveness at different scales.

An additional disparity is observed when considering the specific focus versus the breadth of conditions addressed in studies of skin lesion diagnosis. While some studies have a narrow focus on melanoma and non-melanoma lesions (29), others adopt a broader approach, covering various categories of skin diseases (36). This distinction highlights the decision between targeting a specific condition or taking a more comprehensive approach, which directly influences the clinical applicability of the developed models.

However, there are less ideal scenarios to consider. Afifi et al. (26) and Ramlakhan and Shang (25) use clinical images without specifying their origin, which can negatively impact data quality and representativeness. Additionally, Thissen et al. (27) relies on images obtained from a commercial application, potentially resulting in limitations regarding image quality and diversity. The absence of specification of image origin in Francese et al. (37) is also a factor that can influence data quality and validity.

Finally, the study phase also presents divergences, with some studies still ongoing (24), while others already have final results. The preliminary nature of ongoing studies may limit the availability of conclusive results and the validity of analyses.

It is essential to recognize that both the quantity and type of data play crucial roles in the development of accurate and reliable cutaneous diagnostic models. Larger and more diverse datasets, coupled with high-quality images and reliable sources, tend to produce more robust and generalizable results. Therefore, the careful selection of these elements is fundamental to the effectiveness and clinical applicability of the developed models.

### 3.3 Techniques and processing

Next, we describe the resources employed in image processing, the classification algorithms used, and the devices on which these approaches were implemented. The resources employed in image processing are used to perform manipulation and feature extraction operations, aiming to prepare the images for analysis. Classification algorithms play the role on categorizing skin lesions based on the extracted features, enabling the precise identification of different classes. Furthermore, these algorithms can assist in clinical decision making, guiding healthcare professionals in choosing the best treatment approaches. These approaches are implemented on devices such as computers, servers, or mobile devices, providing efficient execution of algorithms and practical application of diagnostic techniques on skin lesion images.

Extracting this information from the studies presented here is crucial to guide the development of effective applications, allowing the appropriate selection of preprocessing methods, reliable classifiers, and suitable devices for achieving accurate detection and clinical assessment of skin lesions. Table 2 describes the resources used in image processing, the classification algorithms used, and the main purpose of the study.

**Table 2:** Summary of techniques and classifiers used in skin cancer image analysis studies for clinical settings by year and author.

**References**	**Resources used**	**Classifier**	**Purpose**
Ramlakhan and Shang (25)	Image segmentation, feature calculation, and classification.	K-Nearest Neighbors (K-NN).	Classify malignant and benign lesions.
Afifi et al. (26)	Pre-processing, segmentation, feature extraction, and classification.	Support Vector Machine (SVM)	Melanoma detection.
Thissen et al. (27)	Lesion area, mean grayscale value, standard deviation over the lesion, and lesion circularity extracted from fractal map.	The evaluation algorithm is based on fractal and classical image.	Classification of low, medium, or high-risk lesions (where proven benign skin lesions should fall into the low or medium-risk class, and melanoma and non-melanoma skin cancer, along with melanoma *in situ*, actinic keratosis, and Bowen's disease, should fall into the high-risk class).
Roy et al. (28)	Image segmentation, feature calculation, and classification.	YOLOv2.	Melanoma detection.
Alizadeh and Mahloojifar (29)	Pre-processing, segmentation, lesion detection, and classification algorithms.	Normal Bayes and Support Vector Machine (SVM).	Melanoma detection.
Fujisawa et al. (30)	Pre-processing and feature extraction.	GoogLeNet DCNN deep convolutional neural network (DCNN).	Classify malignant and benign lesions.
Udrea et al. (31)	Pre-processing, segmentation, and feature extraction.	Conditional generative adversarial network to segment skin lesions in images. For classification, Support Vector Machine Classifier with radial basis kernel function was used.	Detection of (pre)malignant and malignant conditions.
Bakheet and Al-Hamadi (32)	Image pre-processing, skin lesion segmentation, feature extraction, and classification.	Multilevel Neural Network (MNN)	Melanoma detection.
Abbas (33)	Image pre-processing, skin lesion segmentation, feature extraction, and classification.	The Smart-Dermo system is proposed in this article using image processing and applies clinical rules using the ABC clinical technique. It also uses Fuzzy technique for classification.	Melanoma detection.
Bakheet and El-Nagar (34)	Image pre-processing, adaptive lesion segmentation, and feature extraction.	Deep Neural Network (DNN).	Classification of malignant vs. benign lesions.
Dulmage et al. (35)	Not specified	Deep convolutional neural network (CNN) architecture, including DenseNet and NASNetMobile, as well as proprietary models developed by VisualDx.	Detection of skin lesion morphology.
Pangti et al. (36)	Pre-processing and image optimization resources, normalization algorithms, and custom loss function for training the neural network.	Convolutional Neural Networks (CNN).	Detection of 40 common skin diseases.
Giavina-Bianchi et al. (17)	Similarity networks and Data Augmentation.	In the clinical model, image features are extracted through a convolutional network (VGG16), and then the K-Nearest Neighbor (KNN) algorithm is used to classify the images based on these features. In the dermatoscopic model, images are processed using generative adversarial networks (GANs), and classification is performed through an ensemble model that combines the results of five EfficientNetB6 models.	Melanoma detection.
Francese et al. (37)	Real-time analysis process of skin lesions involves acquiring camera frames, tracking device position relative to the patient's skin, cropping the nevus, image pre-processing, feature extraction, nevus classification using a CNN, pose estimation, rendering, and displaying augmented images.	Convolutional Neural Network (CNN)	Melanoma detection.
Felmingham et al. (24)	Not specified.	Convolutional Neural Network (CNN) developed by MoleMap Ltd and Monash eResearch.	Classification into benign, uncertain, or malignant lesions.
Sangers et al. (38)	Not specified.	The study used a mobile health app called SkinVision, which utilizes Convolutional Neural Network (CNN).	Classification into suspicious and benign lesions.
Jahn et al. (39)	Not specified.	The study used a mobile health app called SkinVision, which utilizes Convolutional Neural Network (CNN).	Melanoma detection.
Kränke et al. (40)	Not specified.	Two CNNs: one classical CNN and the other region proposal network (RPN)-based CNN for stratification.	Classification of various skin lesions.

It is notable that several studies aim to utilize image segmentation, feature extraction, and classification techniques, as observed in Ramlakhan and Shang (25), Afifi et al. (26), Roy et al. (28), Alizadeh and Mahloojifar (29), Bakheet and Al-Hamadi (32) and Abbas (33). These steps are often fundamental for proper processing of skin lesion images and subsequent diagnostic decision-making.

On the other hand, there are differences regarding the choice of classifiers and processing devices. While some studies, such as Afifi et al. (26), employ Support Vector Machines (SVM) as classifiers, others, like Roy et al. (28), opt for more recent approaches like YOLOv2. The research by Roy et al. (28), Bakheet and Al-Hamadi (32), and Giavina-Bianchi et al. (17) presents a variety of approaches, ranging from the use of traditional machine learning algorithms to deep neural networks, such as Convolutional Neural Networks (CNNs). This diversity of techniques allows for a rich comparative analysis, enabling the identification of the most promising approaches for skin tumor detection. Additionally, the detailed description of the resources used and processing devices provides valuable insights for the development of effective applications.

Regarding processing devices, there is a distinction between approaches that perform detection and classification directly on mobile devices, such as Alizadeh and Mahloojifar (29), and approaches that send extracted features to a server for further analysis, as in the case of Giavina-Bianchi et al. (17). This difference highlights the variety of options available for implementing skin lesion detection solutions.

Finally, some studies do not provide complete information about the resources used, such as Dulmage et al. (35), which limits the understanding of the methodologies employed.

### 3.4 Main results and perspectives

In this section, we present the main outcomes and prospective insights derived from the various studies analyzed. The primary classification results demonstrate the accuracy, sensitivity, and specificity achieved by different approaches, allowing an assessment of how reliable these methods are in detecting malignant and benign lesions. Furthermore, the perspectives highlight the unique contributions of each study, such as the use of deep learning algorithms, real-time detection effectiveness, and the potential for screening in populations with limited access to dermatologists.

In the context of medicine and healthcare, this information assists medical professionals in choosing the most suitable approaches for early detection of malignant skin lesions, contributing to more precise and rapid diagnosis. Additionally, these results and perspectives also have significant implications for the future development of healthcare applications, guiding research and innovations in the field of artificial intelligence applied to dermatology.

Table 3 provides details related to the main results and perspectives.

**Table 3:** Overview of classification results and potential implications of skin cancer studies for clinical settings by year and author.

**References**	**Key classification results**	**Prospects**
Ramlakhan and Shang (25)	Sensitivity of 80.5% for benign lesions and 60.7% for malignant lesions.	Demonstrates the ability to perform image segmentation, calculate features, and classify lesions on a smartphone with good recognition accuracy.
Afifi et al. (26)	No results presented regarding the classifier.	The system can detect melanoma in real-time with high accuracy and low power consumption, proposed for use in primary care settings, using a high-level hardware design methodology to implement the SVM classifier quickly and efficiently on an FPGA.
Thissen et al. (27)	Achieved 80% sensitivity and 78% specificity in detecting (pre)malignant conditions.	The evaluated app can support less experienced professionals in differentiating between benign and malignant lesions. It analyzes data related to texture, color, geometric features extracted from images, as well as lesion characteristics (lesion age, pain, itching, bleeding, among others).
Roy et al. (28)	The proposed model, YOLOv2, achieved an average precision of 0.89, average recall of 0.91, overall accuracy of 86.00%, recall of 86.35%, specificity of 85.90%, and a frame rate of 21 FPS, indicating high precision and recall in detecting melanoma in dermoscopic images, as well as efficiency in terms of time.	YOLOv2 is presented as a more efficient and accurate approach than other works in automatic melanoma detection in dermoscopic images. The model can process images in real-time with high precision and recall in melanoma detection, and it is invariant to the presence of hair in the images.
Alizadeh and Mahloojifar (29)	Average accuracy, sensitivity, and specificity were 95%, 98%, and 92.19%, respectively.	Development of a mobile application for skin lesion detection using image processing and machine learning techniques.
Fujisawa et al. (30)	The overall accuracy of the trained DCNN was 76.5%. The DCNN achieved a sensitivity of 96.3% (correctly classified as malignant), and a specificity of 89.5% (correctly classified as benign). Although the accuracy of malignancy classification by certified dermatologists was statistically higher than that of dermatology trainees (85.3% ± 3.7% and 74.4% ± 6.8%, P < 0.01), the DCNN achieved higher accuracy.	Classifying skin tumor images into 14 different diagnoses with higher accuracy than certified dermatologists. However, the authors state that it should be validated in a prospective clinical study before considering its use for screening in general medical practice.
Udrea et al. (31)	The machine learning-based skin lesion risk classification algorithm showed sensitivity of 95.1% for melanoma detection and 90.2% for basal cell carcinomas and squamous cell carcinomas. The algorithm's specificity was 78.3% for melanomas and 92.0% for basal cell carcinomas and squamous cell carcinomas. The overall accuracy of the algorithm was 86.1% for melanomas and 79.0% for basal cell carcinomas and squamous cell carcinomas. The study also showed that the algorithm's performance was consistent across different mobile devices and user groups. Additionally, the study demonstrated that the smartphone app could be a useful tool for skin lesion screening in populations with limited access to dermatologists.	Evaluates the accuracy of the latest version of a smartphone app for skin lesion risk assessment and provides an accessible and user-friendly screening tool for individuals with limited access to dermatologists.
Bakheet and Al-Hamadi (32)	The method achieved an area under the ROC curve (AUC) of 0.94, indicating good performance in distinguishing between benign and malignant lesions. Additionally, the method showed sensitivity of 100%, specificity of 95-99%, positive predictive value (PPV) of 86-90%, and negative predictive value (NPV) of 100%.	Developing an effective and fast method with promising performance and 100% sensitivity. The premise is that the detection of malignant melanoma in skin lesion images can be improved through image processing and machine learning techniques. The proposed method uses specific lesion features, such as color and asymmetry, to classify lesions as benign or malignant.
Abbas (33)	The proposed Smart-Dermo achieved 92% accuracy in classifying malignant melanomas and benign tumors.	The Smart-Dermo app aims to assist dermatologists and healthcare professionals in diagnosing skin lesions, enabling early detection and patient monitoring for skin cancer risk. The work is based on using smartphones as processing devices and training the machine learning algorithm with a database of pre-classified dermoscopy images.
Bakheet and El-Nagar (34)	The method achieved an average accuracy rate of 97.5%, sensitivity of 96.67%, and specificity of 100.0% on a dermoscopy image dataset.	The study promises an efficient and real-time approach for melanoma detection in dermoscopy images, with results comparable to or superior to state-of-the-art methods. The work's premises include using a well-established dermoscopy image dataset and validating the proposed method on a test set.
Dulmage et al. (35)	The main results of the study show that the AI system can categorize skin lesion morphology with 68% accuracy. When considering the top three classifications predicted by the AI system, accuracy increases to 80%. Additionally, the study reveals that the AI system performed similarly to primary care physicians who used visual guidance to assist in lesion morphology categorization.	The work aims to develop an AI system capable of categorizing skin lesion morphology with high accuracy, which can be useful for primary care and emergency physicians in diagnosing skin diseases.
Pangti et al. (36)	The machine learning model achieved an overall accuracy of 76.93% (±0.88%) in top-1 and an average area under the curve (AUC) of 0.95 (±0.02) on clinical images in an *in silico* validation study. In a clinical study with patients of color, the app achieved an overall accuracy of 75.07% (95% CI = 73.75-76.36) in top-1, 89.62% (95% CI = 88.67-90.52) in top-3, and an average AUC of 0.90 (±0.07).	The model was trained on a large dataset of skin lesion images and evaluated in three different clinical settings, including an internal validation dataset, an external validation dataset, and a multicenter prospective clinical study, providing a diagnostic tool for 40 types of skin lesions.
Giavina-Bianchi et al. (17)	Dermoscopy models achieved an accuracy of 89.3% for melanoma, while the clinical model achieved an accuracy of 84.7%. Sensitivity for these models was 0.91 and 0.89, and specificity reached 0.89 and 0.83, respectively. Both models demonstrated a remarkable area under the curve (AUC) exceeding 0.9.	Developed a mobile application with a data collection protocol (photos, demographic information, and brief medical history) and AI to classify clinical and dermoscopic images. The app generates reports for each lesion with images, indicative heatmaps, estimated probability of melanoma or malignancy, likely diagnosis, and management suggestions.
Francese et al. (37)	The results are related to the usability of the application: clarity of tasks (100% of dermatologists found tasks clear), ease of use of the app (5 dermatologists found it easy to use), the need for technical support (100% of dermatologists felt they would not need support), and integration of system functions (100% of dermatologists found functions well-integrated).	It is possible to identify that the work proposes a system for skin lesion analysis that uses augmented reality and deep learning techniques to assist dermatologists in diagnosing skin lesions. The system was evaluated through a post-test questionnaire answered by dermatologists, and the results indicated that the system is easy to use and does not require additional technical support.
Felmingham et al. (24)	The study is still ongoing.	The promises and premises of the work are to assess the effectiveness of the CNN in assisting physicians in diagnosing and managing skin lesions in a real-world clinical environment. The study also aims to evaluate the safety of the AI algorithm before its use in post-intervention settings and assess the acceptance of the AI algorithm by physicians and patients.
Sangers et al. (38)	The app showed an overall sensitivity of 86.9% and specificity of 70.4%. Sensitivity was significantly higher on iOS devices compared to Android devices (91.0% vs. 83.0%). Furthermore, specificity was considerably higher for control benign lesions compared to suspicious skin lesions (80.1% vs. 45.5%). It was also observed that sensitivity was higher in skin fold areas compared to smooth skin areas (92.9% vs. 84.2%), while specificity was higher for lesions in smooth skin areas (72.0% vs. 56.6%).	The study evaluated the effectiveness of the app in detecting skin lesions at risk of skin cancer and concluded that the app has the potential to help patients assess their skin lesions before consulting a healthcare professional.
Jahn et al. (39)	The study assessed the diagnostic accuracy of the SkinVision^®^ smartphone app in melanoma detection and found that the app classified a significantly higher number of lesions as high-risk compared to dermatologists, potentially leading to unnecessary excisions. Additionally, the diagnostic performance of the app was below the advertised rates, with low sensitivity and specificity.	The text highlights the importance of evaluating apps for certification with real-world prospective evidence.
Kränke et al. (40)	The detection algorithm showed a sensitivity of 96.4% and specificity of 94.85%, while the analysis algorithm achieved a sensitivity of 95.35% and specificity of 90.32%.	To evaluate the accuracy of two new neural networks for diagnosing skin cancer on currently available smartphones. The study also aimed to provide a low-cost and easily accessible screening tool for early skin cancer detection.

The analysis of Table 3 highlights the positive aspects of recent advances in the detection, classification, and evaluation of skin cancer applications using machine learning and image processing, achieving high sensitivity and specificity in identifying malignant lesions. Furthermore, mobile applications offer an accessible approach to screening in populations with limited access to dermatologists.

However, more robust clinical validation is needed, considering the testing stage and comparison with traditional diagnosis. Performance variation between devices and the possibility of unnecessary excisions are also issues to be addressed. These advancements represent significant potential, but it is essential to balance opportunities with challenges, prioritizing ongoing research and validations for effective implementations in medical practice.

Among the studies presented, the YOLOv2 model, proposed by Roy et al. (28), stands out by demonstrating high precision and sensitivity in the detection of melanoma in dermoscopic images, processing in real-time efficiently. Additionally, Udrea et al. (31) present a machine learning-based method that achieves significant results in sensitivity and specificity for the detection of melanomas and basal cell carcinomas and squamous cell carcinomas. In turn, Giavina-Bianchi et al. (17) develop dermatoscopy models to assist dermatologists, offering positive perspectives for improving the detection and management of skin lesions. Furthermore, an innovative approach by Francese et al. (37) uses augmented reality and deep learning in a lesion analysis system, with the potential to facilitate dermatological diagnosis.

It is important to note that, although all the approaches highlighted in Table 3 show promising results, many of them are still undergoing testing and clinical validation phases. Therefore, it is crucial to continue rigorous research and in-depth evaluations, as emphasized by various researchers, before considering the widespread and effective implementation of these approaches in medical practice. These innovations have the potential to revolutionize early detection and diagnosis of skin cancer, but ensuring their reliability and clinical utility through robust studies is fundamental.

### 3.5 Diversity, accessibility, and medical collaboration

Ethnic diversity, the involvement of medical professionals, and ethical considerations play a pivotal and indispensable role in the development of applications designed for the detection and classification of skin lesions. These factors significantly contribute to the efficacy, validity, and accessibility of these technological solutions, thereby ensuring their widespread acceptance and adoption within the medical community, characterized by both confidence and equity. The continuous advancement within this scientific domain necessitates a multidisciplinary approach that seamlessly amalgamates the expertise of dermatologists, data scientists, and healthcare practitioners with the overarching objective of further enhancing the precision and impact of these pioneering applications.

Within this context, the systematic incorporation of a comprehensive array of ethnicities and genotypes into the training and evaluation datasets assumes fundamental importance. This strategic inclusion is essential to ensure the capability of such applications to meticulously identify and classify lesions across diverse skin types. This strategic approach contributes profoundly to the reduction of potential biases and affirms the technology's reliability for a broad and variegated spectrum of end-users.

Additionally, the active involvement of seasoned healthcare professionals plays a critical role in the formulation of the training parameters for AI models and the meticulous review of the decisions emanating from these applications. This collaborative synergy serves as an anchor to guarantee diagnostic precision while also facilitating the identification of intricate cases that may pose challenges to the technology. Furthermore, the validation of these applications by dermatologists is of paramount importance in the comprehensive evaluation of their effectiveness in comparison to conventional diagnostic methodologies.

In this manner, Table 4 presents a repository of pertinent information pertaining to the ethnic and genetic diversity of the study population, in conjunction with a meticulous assessment of the participation levels of dermatologists and other healthcare professionals in each research study.

**Table 4:** Diversity considerations and medical professional involvement in skin cancer studies for clinical settings by year and author.

**References**	**Ethnic and genetic diversity of the population**	**Participation of dermatologists and other medical professionals**
Ramlakhan and Shang (25)	Does not present data on this aspect.	Does not present data on this aspect.
Afifi et al. (26)	Does not present data on this aspect.	Does not present data on this aspect.
Thissen et al. (27)	Does not present data on this aspect.	The text mentions that consecutive patients were seen by both a dermatologist and a dermatology resident.
Roy et al. (28)	Does not present data on this aspect.	Does not present data on this aspect.
Alizadeh and Mahloojifar (29)	Does not present data on this aspect.	The text mentions that results are displayed to dermatologists on smartphones, suggesting that the system may be used by healthcare professionals.
Fujisawa et al. (30)	The study mentions that it was conducted in the Division of Dermatology at the University of Tsukuba Hospital but does not provide additional information about the studied population.	The authors compare results with interns and dermatologists, implying that the system may be developed to assist medical professionals in their diagnoses.
Udrea et al. (31)	The data primarily come from countries such as the United Kingdom, the Netherlands, Australia, and New Zealand. However, it does not provide additional information about the studied population's diversity.	Yes, the study mentions that each pair of image and corresponding risk classification undergoes a quality control check performed by a dermatologist. Moreover, for lesions classified as high-risk or for cases that have been upgraded or downgraded by a dermatologist, the user will receive a message from the Customer Care team within 48 hours, indicating the level of urgency. This indicates that there is dermatologist support and involvement in the skin lesion assessment process.
Bakheet and Al-Hamadi (32)	Does not present data on this aspect.	The study mentions that the methodology was developed in collaboration with dermatologists and other medical professionals.
Abbas (33)	Does not present data on this aspect.	The Smart-Dermo application was developed to assist dermatologists and healthcare professionals in diagnosing skin lesions. However, it does not provide detailed information about the specific support of dermatologists and other medical professionals during the application's development. It can be inferred that the application aims to provide an additional tool to assist healthcare professionals in diagnosing and monitoring patients at risk of developing skin cancer.
Bakheet and El-Nagar (34)	Does not present data on this aspect.	Does not present data on this aspect.
Dulmage et al. (35)	The study mentions concerns about the potential for artificial intelligence technology to exacerbate health inequalities among patients of different ethnicities but does not provide specific data on the ethnic and genetic diversity of the studied population. Additionally, the images were classified by Fitzpatrick skin type and separated into darker skin types (Fitzpatrick skin type IV - VI) and lighter skin types (Fitzpatrick skin type I - III).	The study mentions that the artificial intelligence system was developed in collaboration with dermatologists and other medical professionals. The study also mentions that skin lesion images used to train the system were manually labeled by dermatologists.
Pangti et al. (36)	The work mentions the scarcity of clinical images (macroscopic) from different ethnicities as one of the major challenges in developing deep learning-based skin disease classifiers. Additionally, the text mentions that using locally generated data helped address the issue of class imbalance and racial bias in public datasets. However, the text does not provide specific information about the ethnic and genetic diversity of the population used.	Dermatologists were involved in the study to assess the accuracy of the skin disease diagnostic application compared to human dermatologists.
Giavina-Bianchi et al. (17)	Does not present data on this aspect.	Data was obtained for a teledermatology project, meaning it utilized data collected by dermatologists.
Francese et al. (37)	Does not present data on this aspect.	The system was evaluated by dermatologists through a post-test questionnaire.
Felmingham et al. (24)	Does not present data on this aspect.	The study is led by dermatologists and involves other medical professionals, including pathologists and nurses.
Sangers et al. (38)	Does not present data on this aspect.	A set of 239 cases were confirmed through dermatological evaluation and/or histopathology.
Jahn et al. (39)	Does not present data on this aspect.	Seven dermatologists participated in the study as evaluators of the lesions.
Kränke et al. (40)	Does not present data on this aspect.	The study was conducted by the Department of Dermatology at the Medical University of Graz, Austria, suggesting the involvement of dermatologists and other medical professionals in the study's execution.

The studies present diverse approaches in their research endeavors. For instance, Udrea et al. (31) emphasizes the inclusion of data origin information, indicating that the data predominantly comes from countries such as the United Kingdom, the Netherlands, Australia, and New Zealand. On the other hand, Pangti et al. (36) mentions the scarcity of clinical images from different ethnicities as a challenge but addresses this issue by using locally generated data to mitigate class imbalance and racial bias in public datasets.

Another notable difference lies in the validation approach. While Fujisawa et al. (30) and Pangti et al. (36) highlight comparative validation with diagnoses performed by healthcare professionals, Francese et al. (37) focuses on evaluation by dermatologists through post-test questionnaires. Each of these studies adopts a unique strategy to verify the effectiveness and accuracy of the applications.

Moreover, Dulmage et al. (35) draws attention to image classification based on the Fitzpatrick skin type, emphasizing specific considerations for variations in skin tone in their assessments. Conversely, Bianchi et al. (17) utilizes data collected through teledermatology for their project, highlighting a different data acquisition approach.

In summary, the studies exhibit differences in terms of data origin, validation strategies, considerations regarding ethnic diversity, and specific data collection approaches, showcasing the diversity and innovation in the approaches taken to create skin lesion detection applications. However, a central characteristic is the close collaboration with dermatologists and medical professionals, as evidenced in multiple studies. This direct interaction ensures the clinical validity of the applications by aligning the AI decisions with specialized medical knowledge.

Furthermore, comparing results with assessments by dermatologists reinforces the diagnostic accuracy of these technologies. Notably, the explicit consideration of ethnic and genetic diversity within the population, as discussed in Fujisawa et al. (30) and Pangti et al. (36), also stands out as a signigficant strength. By encompassing various skin types and demographic characteristics, such applications become more comprehensive and reliable in real-world scenarios. Taken together, these aspects underscore the relevance of these applications in medical practice and their potential to significantly contribute to early and accurate skin lesion detection.

When analyzing the studies, a consensus becomes evident regarding the importance of accessibility and availability of systems and applications for skin lesion detection and classification. However, many systems still fail fully meet these requirements due to resource limitations, technical complexity, or the absence of clear guidelines. To address this issue, broader collaboration among companies, accessibility experts, programmers, and users is crucial in translating intentions into practical actions. Such collaborative effort will result in significant benefits for all parties involved.

Finally, it is essential to ensure that AI applications are developed and tested ethically and responsibly. This includes safeguarding patient data privacy and security, as well as ensuring transparency in the of development and training processes of algorithms.

## 4 Conclusion

The application of artificial intelligence in dermatology has the potential to revolutionize the detection and diagnosis of skin lesions, especially in the case of melanoma, a severe and potentially fatal disease.

This review highlights that several studies are making significant advancements in improving image processing capabilities, pattern recognition, and deep learning. These advancements enable rapid and accurate analyses that can lead to real-time diagnoses. This evolution contributes to early detection of skin cancer, expanding the prospects for cure and minimizing the reliance on invasive procedures.

However, it is important to note that the vast majority of the solutions presented have not yet been validated in clinical settings or developed in collaboration with dermatologists and other healthcare professionals to ensure they meet patients' needs and are effective in clinical practice.

In summary, the solutions presented can help enhance the efficiency of healthcare services, reducing the time required for examinations and diagnoses. This can be especially important in areas with a shortage of healthcare professionals or in emergency situations where time is critical. However, they should be used with caution and responsibility, in collaboration with dermatologists and other healthcare professionals, to ensure they meet patients' needs and are effective in clinical practice.

## Data availability statement

The original contributions presented in the study are included in the article/supplementary material, further inquiries can be directed to the corresponding author.

## Author contributions

BF: Writing—original draft, Writing—review & editing. BO: Writing—review & editing. RP: Writing—review & editing. JP: Writing—review & editing. RL: Writing—original draft, Writing—review & editing. WC: Writing—review & editing. MR: Writing—review & editing. MG-B: Writing—original draft, Writing—review & editing.
